# The novel immunomodulatory biologic LMWF5A for pharmacological attenuation of the “cytokine storm” in COVID-19 patients: a hypothesis

**DOI:** 10.1186/s13037-020-00248-4

**Published:** 2020-05-13

**Authors:** Gregory Thomas, Elizabeth Frederick, Melissa Hausburg, Laura Goldberg, Marshall Hoke, Michael Roshon, Charles Mains, David Bar-Or

**Affiliations:** 1 Ampio Pharmaceuticals, Inc, 373 Inverness Pkwy #200, Englewood, CO 80112 USA; 2 Trauma Research Department, Swedish Medical Center, 501 E. Hampden, Englewood, CO 80113 USA; 3 Trauma Research Department, St. Anthony Hospital, 11600 W 2nd Pl, Lakewood, CO 80228 USA; 4 Trauma Research Department, Medical City Plano, 3901 W 15th St, Plano, TX 75075 USA; 5 Trauma Research Department, Penrose Hospital, 2222 N Nevada Ave, Colorado Springs, CO 80907 USA; 6 Trauma Research Department, Research Medical Center, 2316 E Meyer Blvd, Kansas City, MO 64132 USA; 7 Trauma Research Department, Wesley Medical Center, 550 N Hillside St, Wichita, KS 67214 USA; 8 Emergency Department, Penrose Hospital, Colorado Springs, Colorado USA; 9 Centura Health Systems, Centennial, Colorado USA; 10 Department of Molecular Biology, Rocky Vista University, 8401 S Chambers Rd, Parker, CO 80134 USA; 11 Swedish Medical Center, 501 E. Hampden Ave. Rm 4-454, Englewood, CO 80013 USA

**Keywords:** LMWF5A, Cytokine storm, SARS-CoV-2, COVID-19, Acute lung injury, Acute respiratory distress syndrome, Barrier function

## Abstract

**Background:**

A common complication of viral pulmonary infections, such as in the ongoing COVID-19 pandemic, is a phenomenon described as a “cytokine storm”. While poorly defined, this hyperinflammatory response results in diffuse alveolar damage. The low molecular weight fraction of commercial human serum albumin (LMWF5A), a novel biologic in development for osteoarthritis, demonstrates beneficial in vitro immunomodulatory effects complimentary to addressing inflammation, thus, we hypothesize that LMWF5A could improve the clinical outcomes of COVID-19 by attenuating hyperinflammation and the potential development of a cytokine storm.

**Presentation of the hypothesis:**

A variety of human in vitro immune models indicate that LMWF5A reduces the production of pro-inflammatory cytokines implicated in cytokine storm associated with COVID-19. Furthermore, evidence suggests LMWF5A also promotes the production of mediators required for resolving inflammation and enhances the barrier function of endothelial cultures.

**Testing the hypothesis:**

A randomized controlled trial, to evaluate the safety and efficacy of nebulized LMWF5A in adults with Acute Respiratory Distress Syndrome (ARDS) secondary to COVID-19 infection, was developed and is currently under review by the Food and Drug Administration.

**Implications of hypothesis:**

If successful, this therapy may attenuate the cytokine storm observed in these patients and potentially reduce mortality, increase ventilation free days, improve oxygenation parameters and consequently lessen the burden on patients and the intensive care unit.

**Conclusions:**

In conclusion, in vitro findings suggest that the immunomodulatory effects of LMWF5A make it a viable candidate for treating cytokine storm and restoring homeostasis to the immune response in COVID-19.

## Background

Severe acute respiratory syndrome coronavirus 2 (SARS-CoV-2) is the virus responsible for the infectious respiratory condition now formally known as coronavirus disease of 2019 (COVID-19) [1]. This virus was first identified in Wuhan, Hubei Province, China in December of 2019 and declared a global pandemic by the World Health Organization in March 2020 [2]. COVID-19 symptoms (cough, fever, and shortness of breath) generally appear within 14 days of exposure and 20% of these patients progress to severe disease [3]. Hallmarks and complications of severe COVID-19 infection include acute respiratory distress syndrome (ARDS), pneumonia, sepsis and septic shock, cardiomyopathy and arrhythmia, acute kidney injury, and prolonged hospitalization [3]. Based on the size and scope of the COVID-19 pandemic, the disease burden on healthcare facilities and hospitals is severe, to the point that the US will continue to experience widespread shortages of critical standard of care items such as ventilators [4].

While the pathogenesis of COVID-19 is still poorly defined, it is believed to involve viral-induced suppression of innate pathogen surveillance systems. Under normal conditions, pathogen pattern recognition receptors (PPR) on resident innate immune cells sense viral RNA molecules that trigger anti-viral, interferon (IFN) expression which prevents replication and promotes the removal of infected cells [5]. However, genomic studies conducted on the original severe acute respiratory syndrome coronavirus (SARS-CoV) demonstrate that the virus encodes for proteins that serve as innate immune antagonists by suppressing the expression of IFN and promoting evasion of viral RNA from host defense mechanisms, independent of pro-inflammatory cytokine release [5]. As a result, early in infection, innate toll-like receptor (TLR) and PRR signaling pathways continue to potentiate the release of pro-inflammatory mediators, such as cytokines (i.e., TNFα, IL-6, IP10 or CXCL10, etc.) while viral replication remains unchecked. Hence, it has been theorized, coronaviruses pathogenesis involves the delayed release of IFN and an accumulation of monocyte/macrophages together with an inappropriate T-cell response [6]. Adding support to this etiology, severe cases of COVID-19 appear to present with dysregulated T-cell counts and elevated inflammatory cytokine levels [7].

In the case of severe COVID-19, this disruption leads to a condition described as a “cytokine storm”, in which excessive amounts of pro-inflammatory cytokines are produced and may contribute to morbidity and mortality in these patients. A potential INFγ-related cytokine storm was first identified in some patients suffering with SARS-CoV infection involving a distinct pattern of cytokines including IFNγ, CXCL10, and IL-6 [8]. Subsequently, clinical studies are now confirming similar responses in COVID-19 patients. In a study published in the Lancet, COVID-19 patients exhibit increased plasma levels of cytokines and chemokines, such as IFNγ, CXCL10, IL-1β, and TNFα [9]. As a result, therapeutic interventions for COVID-19 must address a range of pro-inflammatory cytokines and chemokines that can direct the arrival of immune cells and the development of a late phase hyperactivation.

It has been proposed this hyperinflammatory response triggers a violent attack on the body that potentiates cytokine storm development [10]. Studies demonstrate that viral infections, through both the result of viral-induced cellular toxicity and the immune response itself, drive the production of damage-associated molecular pattern (DAMP) molecules that are recognized by innate TLRs. For example, Imai et al. have demonstrated that oxidized phospholipids, generated by reactive oxygen species following exposure to inactivated influenza virus, lead to TLR4-mediated alveolar macrophage cytokine release and acute lung injury (ALI) in mouse models [11]. Importantly, they also found that inactivated influenza virus causes oxidative stress and TLR4 mobilization in human peripheral blood mononuclear cells (PBMC) [11]. In addition, viral infections have been shown to induce TLR4-mediated release of pro-inflammatory cytokines through the release of the DAMP molecules S100 calcium-binding protein A9 and high mobility group box 1; proteins normally sequestered inside the cell [12, 13]. Functionally, blocking TLR4 protects from and TLR−/− mice are highly resistant to influenza-induced lethality [11, 14]. It is also important to note, that TLR4 signaling may contribute to fibrosis, further complicating management of COVID-19 [15]. These observations suggest that DAMP-mediated TLR signaling is an important therapeutic target in COVID-19, to reduce feed-back loops potentially critical for cytokine storm development. Interestingly, T-cells infected with SARS-CoV exhibit elevated expression of TLR4, 7, and 9, further demonstrating that this is a critical target for coronavirus intervention [16].

The low molecular weight fraction of commercial human serum albumin (LMWF5A), a novel biologic drug in development for the treatment of inflammation associated with osteoarthritis, exhibits mechanisms of action that may be complimentary to addressing the innate-immune-mediated inflammation seen in patients suffering from COVID-19. In the course of development history, the biologic effects of LMF5A have been established using a variety of human immune cell ex vivo and in vitro models stimulated using the DAMP, TLR4 agonist lipopolysaccharide (LPS) as well as relevant barrier-function assays using human endothelial cells (Table 1; also see review on LMWF5A mechanisms of action [25]). Thus, we hypothesize that LMWF5A could improve the clinical outcomes of COVID-19 by attenuating hyperinflammation and the potential development of a cytokine storm as well as the resulting increase in vascular permeability. In this report, we discuss key findings that support the use of LMWF5A as a therapeutic agent for patients suffering from COVID-19 following and provide translational links drawn from literature searches to bridge our research to modulation of key inflammatory mediators and function of the alveolar-epithelial barrier.

**Table 1:** Ex vivo and in vitro immunomodulatory and barrier function effects of LMWF5A

Cell Model	Results	Conclusions	Study
Influenza HA presented human T-cell clone	LMWF5A and DA-DKP treatment results in: ↓ TNFα ↓ IFNγ	Reduced release of cytokines associated with COVID-19 cytokine storm.	[17]
Influenza HA presented and CD3/CD28 stimulated human T-cell clone	DA-DKP treatment results in: ↓ TNFα ↓ IFNγ ↑ RAP-1 phosphorylation and activity	Reduced release of cytokines associated with COVID-19 cytokine storm. Also, increased activation of barrier enhancing GTPase.	[18]
LPS-stimulated human PBMC	LMWF5A and DA-DKP treatment results in: ↓ TNFα	Reduced release of cytokine associated with COVID-19 cytokine storm.	[19]
LPS-stimulated human PBMC	LMWF5A treatment results in: ↓ TNFα ↑ PGE2 and 15d-PGJ2	Reduced release of cytokine associated with COVID-19 cytokine storm together with increased pro-resolving mediator release.	[20]
LPS-stimulated, PMA-differentiated THP-1 macrophages	LMWF5A treatment results in: ↓ IL-6, IL-12, and CXCL10 ↑ IL-10 ↑ AhR activity	Reduced release of cytokines associated with COVID-19 cytokine storm with apparent shift from M1 to M2 phenotype.	[21]
Monolayer and 3D cultured human BMMSC	LMWF5A treatment results in: ↓ RhoA activity ↑ Rac1 activity ↓ Stress fiber formation ↑ Stem cell homing potential	Rebalancing of overall GTPase activity conducive to barrier enhancement. Also, increased progenitor cell homing potential.	[22]
Dedifferentiated primary human chondrocytes	LMWF5A treatment results in: ↑ SRY-Box transcription factor ↓ Apoptosis	Activation of transcription factor protective of fibrosis and increased cell survival.	[23]
Primary human endothelial cell permeability models	LMWF5A treatment results in: ↑ Acetylation of α-tubulin ↓ Vascular leakage	Enhanced barrier function of endothelial cells with reduced vascular leakage. Also, apparent stabilization of microtubule network.	[24]

### Presentation of the hypothesis

#### LMWF5A inhibits the release of pro-inflammatory cytokines from PBMC, macrophages, and T-cells

**Fig. 1:**
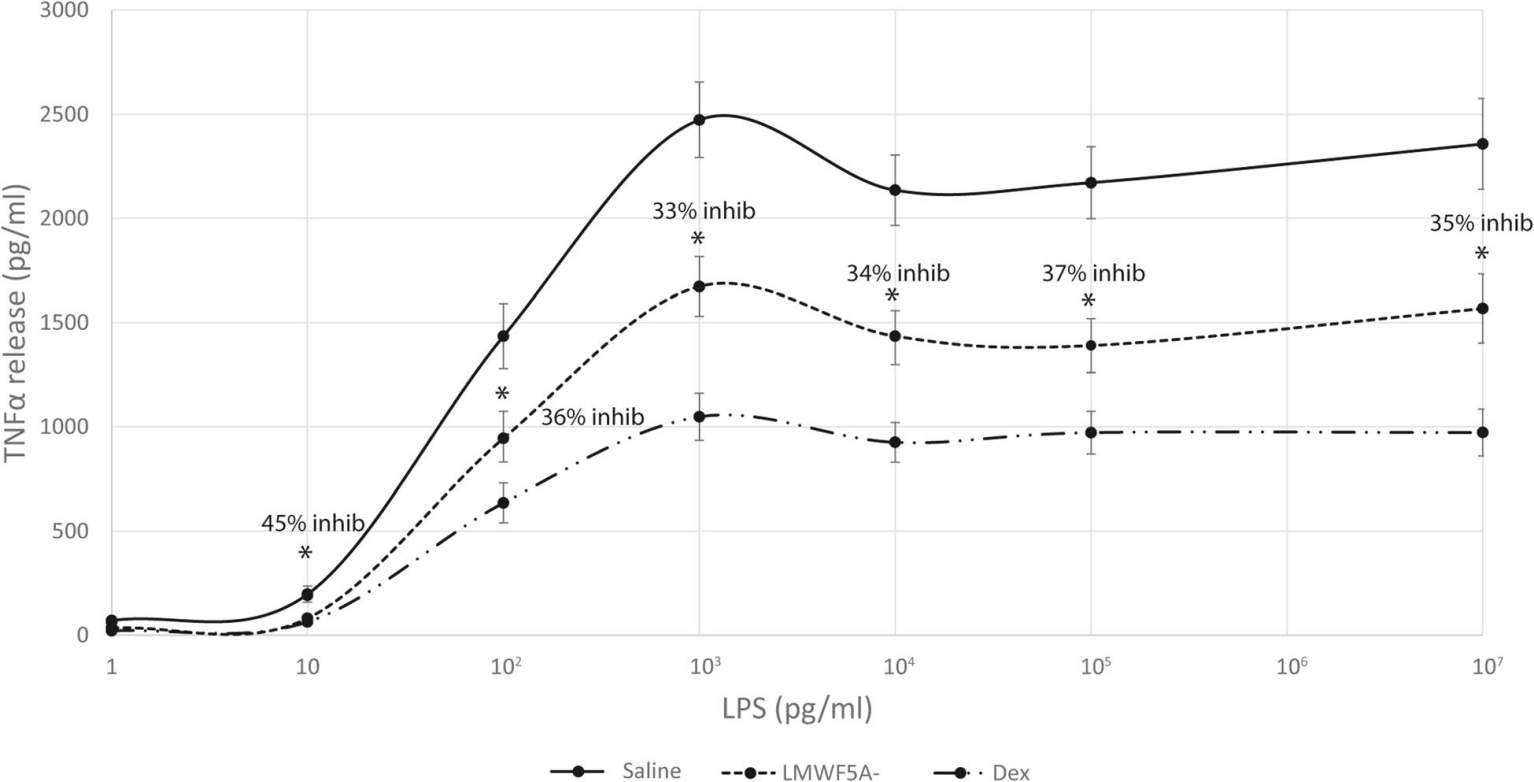
TNF*α* release from LPS-stimulated PBMC in the presence of LMWF5A. PBMC were incubated with LMWF5A, 0.1 μM dexamethasone, or saline for 1 h then stimulated with LPS for 18 h. TNF*α* release was determined by ELISA and presented as means ± SEM from 13 individual donors. % inhibition in LPS-induced TNFα release was also calculated for the LMWF5A treatment groups versus saline control release (∗ = *p* ≤ 0.05 vs. saline control). Adapted and modified from Thomas et al. 2016 [19]

One of the ways LMWF5A may address the excessive cytokine production seen in these patients is by suppressing pro-inflammatory cytokines released from mononuclear cells infiltrating from blood. This is reflected in the ability of LMWF5A to inhibit the release of a key set of cytokines and chemokines associated with SARS-CoV-2 infection in ex vivo human PBMC models activated through innate pattern recognition pathways. For example, LMWF5A reduces TNFα release by human PBMC stimulated using LPS as an agonist for TLR4 signaling (Fig. 1) [19]. LPS-stimulation mimics the pathogen-associated molecular pattern (PAMP) and DAMP signaling seen in the end stage of disease where excessive viral loads and tissue damage trigger excessive induction of the immune response. Subsequent unpublished findings show that this response extends to a reduction of CXCL10, IL-1β, and IL-12 as well. Thus, demonstrates that LMWF5A treatment appears to target the release of a pattern of cytokines observed in COVID-19 and other viral cytokine storms.

Tissue resident and blood-derived macrophages are key contributors in the inflammatory response to viral infections and the pro-inflammatory precursors of ARDS [26]. Alveolar macrophages are the predominant tissue resident immune cells found in the lung and are likely to be involved in both the early anti-viral response and trophic end-stages of tissue damage and recovery. In addition, pleiotropic monocytes, invading across the capillary-epithelial bed, will differentiate into pro-inflammatory M1 macrophages upon arrival and may contribute to the excessive immune response in the lung [26]. Moreover, macrophages that develop into an inflammatory M1 lineage become a potent source of inflammatory cytokines (e.g., TNFα, IL-1β, IL-6, IL-12, CXCL10), furthering inflammation [26, 27]. Of note, the balance of macrophage polarization between the microbial/IFNγ-induced M1 phenotypes and the anti-inflammatory M2 could dictate the amplitude of classical activation versus neutrophil efferocytosis and immune resolution, respectively, during pulmonary insult [28].

In vitro studies using human macrophages further support an immunomodulatory action with LMWF5A treatment by shifting macrophage phenotypes from an inflammatory M1 lineage to an anti-inflammatory M2 lineage [21]. In these experiments, a human THP-1 monocyte cell line was differentiated to induce macrophage-like characteristics, then treated with LMWF5A and stimulated with LPS. Reductions in both secreted cytokine and mRNA transcription were observed for the M1 markers IL-6, CXCL10, and IL-12. Importantly, the same cells exhibited an increase in the release of the anti-inflammatory M2 marker, IL-10 with LMWF5A treatment as compared to saline controls. The reduction in inflammatory M1-type cytokine release and gene expression combined with increased anti-inflammatory M2-type cytokine release indicates LMWF5A modulates the immune response by shifting the cytokine profile towards homeostasis. This shift in macrophage phenotype could reduce macrophage hyperactivity and partially address the overproduction of inflammatory cytokines observed in COVID-19.

Although COVID-19 may be characterized as an innate response, studies also indicate that adaptive cells contribute to the etiology of lung jury as well. Animal models demonstrate that T-cells facilitate the release of pro-inflammatory cytokines, such as TNFα, and the arrival of neutrophils in the lung [29]. As observed with macrophage polarization, this may result from an imbalance in inflammatory and regulatory subsets. For example, the ratio of pro-inflammatory Th17 to T regulatory cytokines in the peripheral blood of patients has been found to be predictive of 28-day mortality with ARDS [30]. In support of this evidence, activated and proliferating pro-inflammatory T-cells have been detected in bronchoalveolar lavage samples taken from ARDS patients [31]. Moreover, lymphocyte counts have been associated with increased disease severity in COVID-19 with patients who die exhibiting significant leukopenia [32]. These cells represent a potentially underappreciated source of both IFNγ and proinflammatory cytokines, such as TNFα, and contributors of disease progression in COVID-19. Once in the lung, INFγ priming of T-cells will provide for intense, superantigen-like signaling that may exacerbate autocrine and paracrine cytokine activity. While their role in COVID-19 is yet to be fully elucidated, targeting persistent and long-lived immune regulators, such as T cells, could prove valuable in balancing the immune response.

One of the earliest documented activities of LMWF5A is its ability to reduce cytokine release from immune cells stimulated through the T-cell receptor (TCR) by CD3/28 antibody or specific antigen presentation. LMWF5A was found to reduce TNFα and IFNγ release from CD3/28-stimulated PBMC and influenza HA antigen-presented human T-cell clones [17]. This activity was attributed, in part, to an identified diketopiperazine molecule formed by the cleavage and cyclization of the two N-terminal amino acids of human serum albumin, DA-DKP, present in LMWF5A. Treatment of these cells with DA-DKP resulted in significant reductions of TNFα and IFNγ release from antigen presented T-cells clones [18]. Interestingly, this effect appears to be specific to memory (CD45RO+) but not naïve (CD45RA+) phenotypes (unpublished findings). These findings demonstrated that LMWF5A can reduce cytokine release during an adaptive immune response triggered by the presentation of antigen or activation of T-cells. Therefore, with respect to COVID-19, LMWF5A may shift the overall inflammatory response away from the hyper-cytokine production observed as the disease transitions to ARDS without impacting the body’s ability to fight the primary viral infection.

#### LMWF5A down-regulates pro-inflammatory transcription factors

Mechanistically, COVID-19 pathogenesis seems to be conducted through distinct transcriptional signaling pathways. For example, activation of NF-κB appears to be a hallmark of alveolar macrophages found in patients during ARDS [33]. More importantly, inhibition of NF-κB has been shown to limit the production of pro-inflammatory cytokines like IL-6 and CXCL10 in mouse models resulting in reduced mortality [34]. Dysregulation of NF-κB activity has been broadly implicated in the production of inflammatory cytokines (TNFα, IL-1β, IL-6) and cell apoptosis [35–37]. Also, it has been reported that NF-κB signaling pathways can coordinate with other hallmark pro-inflammatory transcription factors in the immune response [38].

Another pro-inflammatory transcription factor associated with lung inflammation is signal transducer and activator of transcription (STAT). The canonical IFNγ signaling pathway involves the activation of Janus kinase and STAT1 which promotes M1 polarization of macrophages and augments TCR signaling priming [39]. STAT1 and STAT3 activation has also been shown to be a driving factor in LPS-induced lung injury by mediating the release of IL-6 and TNFα [40]. Supporting this mechanism, inhibition of STAT3 reduces the accumulation of immune cells as well as the amount of detectable TNFα and IL-1β in bronchoalveolar lavage fluid in LPS-induced mouse lung ALI models [41].

Several lines of preliminary evidence demonstrate that LMWF5A reduces the activity of both NF-κB and STAT. NF-κB luciferase HEK293 reporter cells treated with LMWF5A and then stimulated with TNFα exhibit dose-dependent reductions in NF-κB expression as measured by luciferase activity. As for STAT, preliminary experiments, using the LPS-stimulated human PBMC model described above, indicate that LMWF5A reduces STAT1 and STAT3 activation as measured by DNA-binding ELISA in nuclear and cytoplasmic protein fractions taken from cells after 24 h in culture. While these findings need to be fully evaluated, reduction in NF-κB activity could reduce the overall inflammatory status of COVID-19 patients while a reduction in STAT could provide an avenue to help suppress the robust IFNγ and M1 signaling that appears to trigger the cytokine storm development in these patients.

#### LMWF5A upregulates anti-inflammatory transcription factors

Conversely, LMWF5A appears to activate the anti-inflammatory and/or immunoregulatory transcription factors, aryl hydrocarbon receptor (AhR) and peroxisome proliferator-activated receptor (PPAR). A source of endogenous AhR ligands are tryptophan metabolites, and one of the active ingredients in LMWF5A is n-acetyl-tryptophan, suggesting that AhR activation contributes to the immunomodulatory action of LMWF5A. The effect on AhR activity was confirmed using an AhR antagonist (CH223191) in the THP-1 model described above. When an AhR antagonist was added to these macrophage-like cultures, the IL-6 response was partially attenuated, demonstrating that the AhR activation plays a partial role in the reducing of cytokine observed in this model. However, AhR antagonism had no significant effect on CXCL10 inhibition, indicating other pathways are involved in LMWF5A activity as well [21]. Another target pathway for activity was identified based on the fact that endogenous PPAR ligands are comprised of fatty acids and their derivatives, and LMWF5A contains the fatty acid, caprylate. Furthermore, 15-delta prostaglandin J2 (15d-PGJ2), a resolving prostaglandin known to be a natural ligand for PPARγ, is upregulated by LMWF5A (to be discussed more below). To establish the contribution of PPAR in our models, DNA-binding and pathway specific antagonism was evaluated. Preliminary findings suggest that human PBMC treated with LMWF5A exhibited increased PPAR DNA binding and the addition of the PPARγ antagonist GW9662 to these cultures results in the attenuation of the LPS-induced TNFα inhibition. Together, these data indicate that both AhR and PPAR transcriptional pathways play a role in the anti-inflammatory responses observed by LMWF5A.

A large body of evidence suggests that AhR and PPAR signaling plays a pivotal role in immunosuppression and the direction of regulatory immune cell phenotypes. To illustrate, AhR has been shown to suppress NF-κB activity by: increasing DNA binding of the NF-κB subunit p50 (p50)/p50 NF-κB homodimer to competitively inhibit the active p50/ NF-κB subunit p65 (RelA) heterodimer [42], sequestering co-regulators (RelA, NF-κB subunit RelB) to prevent translocation into the nucleus [43] and direct trans-repression in the presence of STAT1 [44]. AhR also promotes anti-inflammatory cytokine production (IL-10, IL-21) and the differentiation of T-cells to regulatory phenotypes through cross-talk with the transcription factors proto-oncogene c-Maf and STAT3 [45, 46]. As with AhR, PPAR has been shown to suppress NF-κB activity by: binding to DNA and directly interacting with the RelA and p50 subunits of NF-κB, inducing the expression of inhibitor NF-κB protein, sequestering coactivators required for NF-κB such as CREB-binding protein, and releasing the repressor B-cell lymphoma 6 protein which redirects its activity towards NF-κB-mediated promoters [47]. This activity decreases the production of several pro-inflammatory cytokines regulated by the NF-κB pathway, including TNFα and IL-6 [47, 48]. More importantly, the PPARγ agonist, rosiglitazone, is protective of endotoxin induced ARDS in rat models with marked reductions in nitric oxide and oxidative damage observed [49]. Based on these results, we suspect that activation of these pathways could help suppress the release of key cytokines, such as IL-6, during COVID-19, provide additional control over pro-inflammatory signaling pathways, and potentially rebalance inflammatory immune cells to regulatory and immunotolerant phenotypes that appear to be missing in critical stages of ARDS.

#### LMWF5A enhances the release of pro-resolving lipid mediators

**Fig. 2:**
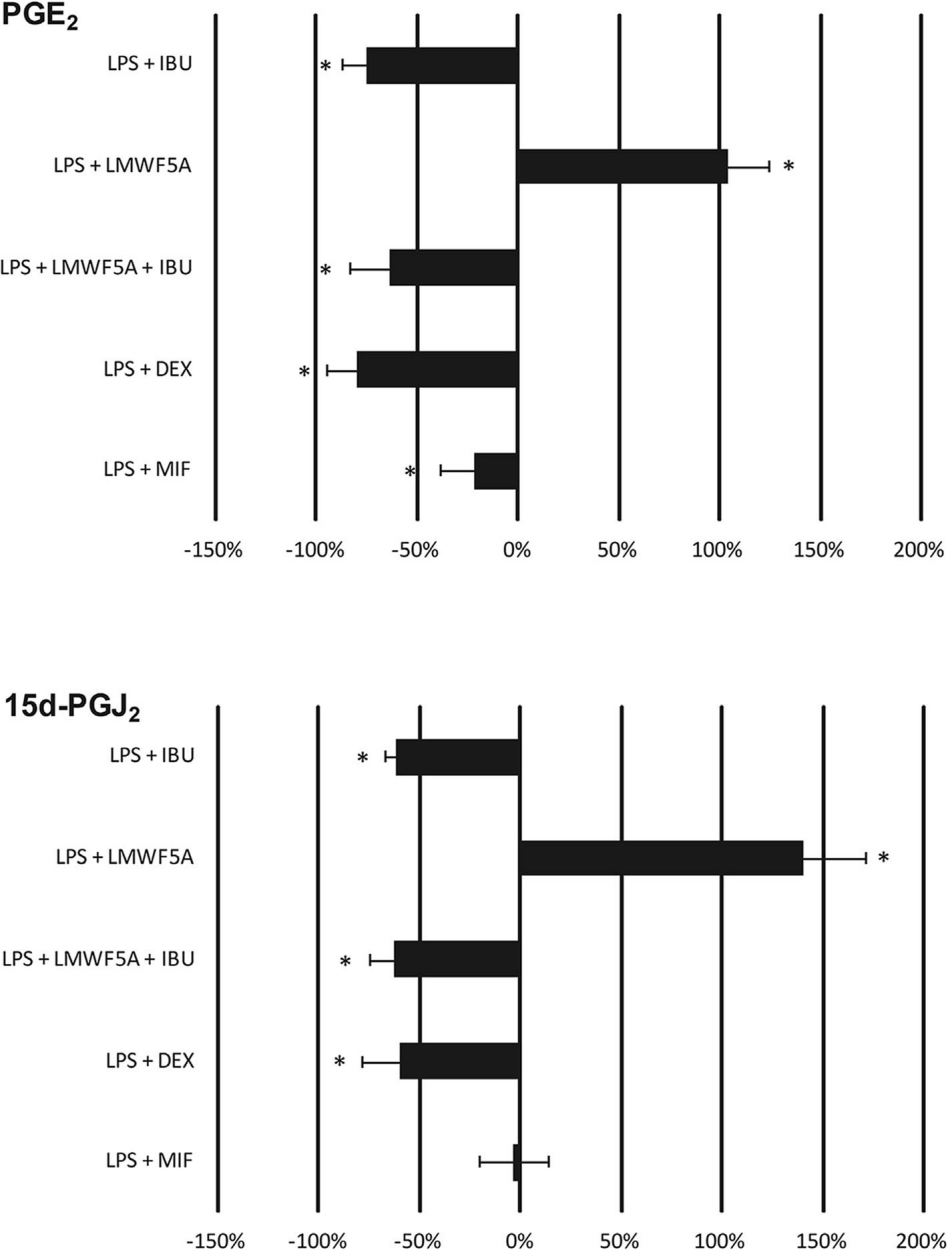
Differential modulation of prostaglandins from LPS-stimulated PBMC pre-treated with LMWF5A and other anti-inflammatory compounds. PBMC were incubated with compounds for 1 h, followed by overnight stimulation with 100 ng/mL LPS. PGE_2_ and 15d-PGJ_2_ release were determined by ELISA, and percent changes are presented as mean ± SD (*N* = 3). (* = *p* < 0.05 versus saline control). Adapted and modified from Thomas et al. 2016 [20]

One of the unique aspects of LMWF5A-induced immunomodulation is the suppression of pro-inflammatory cytokines concomitant with the enhancement of pro-resolving molecules. In our LPS-stimulated PBMC investigations, we found that unlike the steroid dexamethasone, which reduces cytokine and prostaglandin release, LMWF5A inhibits TNFα release while potentiating the production of pro-resolving prostaglandin E2 (PGE2) and 15d-PGJ2 in these cultures (Fig. 2) [20]. On the other hand, treatment with ibuprofen, a nonsteroidal anti-inflammatory drug, strongly attenuates prostaglandin release but does not result in a significant reduction in TNFα release. Consequently, LMWF5A offers a distinctive immunomodulatory profile to other hallmark anti-inflammatory drugs. Western blot analysis shows that LMWF5A accomplishes this by upregulating prostaglandin-endoperoxide synthase 2 (COX-2) expression [20]. A similar PGE2 response is observed in macrophage-like cell culture models using PMA-differentiated U937 monocytic cells stimulated with LPS, suggesting that while LMWF5A can suppress pro-inflammatory cytokines, healing and resolution phase mediators are still produced and possibly enhanced by treatment.

In regard to COVID-19, it is now appreciated that COX-2 plays a critical role in protection through the resolution phase of inflammation. Hangai et al. found that PGE2 release from necrotic macrophages may represent a mechanism to suppress DAMP-induced inflammation and that inhibition of COX-2 results in elevated serum TNFα levels in liver necrotic models [50]. Interestingly, studies have also shown that PGE2 in the wound site is sufficient to drive the reverse migration of neutrophils as well as promote the apoptosis of neutrophils and efferocytosis critical for the resolution phase of the inflammatory process [51]. The authors further hypothesized that this activity is temporally important, in part, to allow neutrophils to perform critical functions, then provide timely resolution. While it is certain that DAMP signaling has evolved to help achieve homeostasis after insult or injury, excessive and/or sustained responses can exacerbate disease. As a result, compensatory mechanisms exist within the biologic milieu and it appears that LMWF5A may help drive lipid-mediated feed-back loops of immunosuppression.

More importantly, this pathway also appears critical to viral immunity and may afford some protection from the robust inflammation in late phase viral infection that leads to ARDS. For example, in some coronavirus studies, it has been shown that the virus manipulates prostaglandin release from infected cells during different phases of infection. Fang et al. showed that SARS-CoV achieves this is by membrane protein-induced down regulation of NF-κB, with a resulting reduction in COX-2 expression [52]. The authors suggest that this is a protective measure by the virus to evade the immune response in the early stages of infection. In addition, the host response with these molecules also appears to dictate disease progression. To illustrate, in mouse studies, age-related differences in PGD2 release result in defective migration of dendritic cells and cytotoxic CD8+ T-cell activity into the lung [53]. Furthermore, it has also been found that anti-inflammatory PGD2 signaling, through D-prostanoid receptor 1, reduces inflammasome-induced IL-1β release and mortality in coronavirus-infected mice [54]. Finally, influenza studies have established that 15d-PGJ2 treatment protects mice against lethal influenza infection through a PPARγ-dependent mechanism with a marked reduction in viral load and lung inflammation observed [55]. It has been theorized that bioactive lipids, such as arachidonic acid, may serve as endogenous anti-viral compounds, thus, together with the findings above, prostaglandins could provide a therapeutic advantage for ARDS secondary to viral infections such as COVID-19 [56].

#### LMWF5A enhances endothelial barrier function

There are many clinical parameters that may contribute to the pathogenesis and morbidity associated with COVID-19. One of these is dysregulation of the alveolar-epithelial barrier, which results in the build-up of protein-rich fluid and decreased oxygen diffusion. Resolution and repair of the widespread inflammation characteristic of ARDS, depends not only on clearance of infiltrating immune cells and suppression of inflammation but also on removal of fluid and restoration of barrier function. Interventions that enhance barrier function should provide a rational approach for the treatment and prevention of COVID-19.

#### Prostaglandins and barrier function

Research findings suggest that LMWF5A may protect barrier integrity in several ways. First, and expanding on an intrinsic ability described above, prostaglandins and COX-2-derived mediators also appear to promote the recovery of barrier function in both endothelial and epithelial cells. To illustrate, PGE2 release from LPS-stimulated A549 epithelial cells, acting through PGE2 receptor EP4, has been shown to enhance microvascular endothelial cell barrier function [57]. Moreover, murine models indicate that COX-2 derived mediators are protective of acid-induced ALI and that selective inhibition delays resolution [58]. Thus, the authors propose that increased COX-2 activity could provide some level of protection against the edema associated with ALI [58].

#### LMWF5A enhances endothelial barrier function

Equally significant, LMWF5A, and its component DA-DKP, have been found to impact the activity of enzymes that link extracellular signals to cytoskeletal rearrangements known as small guanosine triphosphate hydrolases (GTPases). Proteomic analysis and pulldown assays of human T-cells stimulated through the TCR shows that DA-DKP elevates the phosphorylation and activity of the GTPase, Ras-related protein RAP-1 (RAP-1), as compared to controls [18]. Changes in the activity of other GTPases are also observed in bone-marrow derived mesenchymal stems cells (BMMSC) treated with LMWF5A. Following treatment with LMWF5A, BMMSC exhibit a rapid reduction in the intracellular level of active Ras homolog family member A (RhoA) together with an increase in Rac family small GTPase 1 (Rac1) [22]. Finally, DA-DKP treatment of human umbilical vein endothelial cells appears to reduce RhoA activation induced by thrombin (unpublished findings). This body of evidence provides support for the idea that LMWF5A treatment can rapidly regulate GTPase activity in culture.

These molecular switches are intimately linked to a variety of cell processes that help regulate the barrier function of both endothelial and epithelial cells. Of critical importance, specific exchange protein activated by cAMP (EPAC) activation of RAP-1 both prevents and reverses dysregulation of vascular function induced by inflammatory cytokines through stabilization of cytoskeletal components [59]. As seen in endothelial cells, epithelial cell GTPase activity dictates the arrangements and status of f-actin cytoskeletal elements and cellular junction proteins [60]. It is important to note there is relevance of this finding to COVID-19 as studies on the pathology of severe coronavirus-induced ARDS/ALI hypothesize that alveolar damage is dependent on a balance between coagulation and fibrinolysin pathways [61]. Interestingly, viral infection also appears to activate RhoA activity in some models [62]. Because one of the underlying features of ARDS secondary to COVID-19 is the breakdown of the endothelial-epithelial barrier in the alveoli, this activity could provide a potential avenue to reduce the influx of fluid into alveolar spaces.

**Fig. 3:**
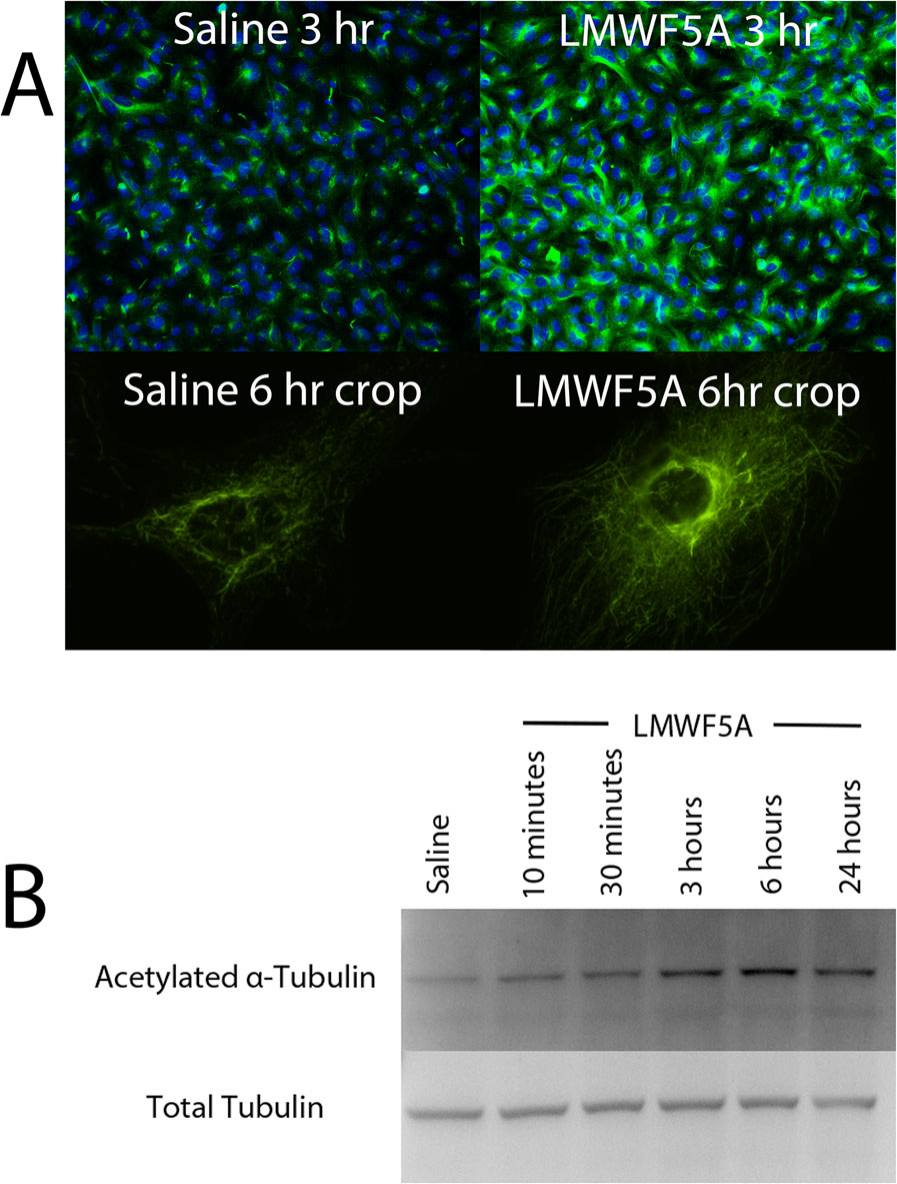
LMWF5A-induces changes in acetylated α-tubulin in HREC. **a**: An increase in total and perinuclear localization of acetylated α-tubulin is observed following LMWF5A treatment. Representative immunofluorescence staining for acetylated α-tubulin in HREC treated with Saline or LMWF5A for 3 or 6 hours (h). Acetylated α-tubulin in saline-treated controls is primarily located in microtubule organizing centers around the nucleus. LMWF5A treated HREC exhibit elevated cytoplasmic and perinuclear staining. Green represents conjugated Alexa fluor 488 anti-acetylated α-tubulin staining and blue represents nuclear 4′,6-diamidino-2-phenylindole DNA staining. **b**: Rapid increase in acetylated α-tubulin is observed following LMWF5A treatment. Western blot analysis of total protein extracts taken from HREC 10 min to 24 h post LMWF5A treatment as compared to total tubulin controls. Adapted and modified from Thomas et al 2016 [24]

An ancillary cytoskeletal rearrangement has also been observed following LMWF5A treatment of human retinal endothelial cells. When treated, these cells observe a rapid increase in acetylated α-tubulin; a modification associated with stabilization and longevity of polymerized microtubules (Fig. 3a) [24]. This post-translational modification, attributed to the release of Ca^2+^ from intracellular stores in this model, can be detected in these cells in as little as 30 min post-treatment and persists for 24 h (Fig. 3b) [24]. To put this in perspective, destabilization of microtubules has been shown to be an integral endothelial barrier dysfunction as a result innate immune signaling and oxidative stress [63]. Moreover, inhibition of histone deacetylase 6, resulting in the acetylation of α-tubulin, has been shown to reduce LPS-induced lung injury by down regulating caspase 1 activity, resulting in lowered IL-1β levels [64]. Ergo, microtubule stabilization has a documented anti-inflammatory and barrier enhancement effect in the lung.

The potential biologic relevance of this LMWF5A activity can be seen in functional assays of barrier activity and in the microscopic examination of cytoskeletal rearrangements. Treatment of both endothelial and epithelial monolayers with DA-DKP increases barrier function for extended periods of time as measured by increased trans endothelial electrical resistance. Moreover, DA-DKP also attenuates rapid increases in permeability, promotes cortical f-actin rearrangement, and reduces stress-fiber formation following thrombin stimulation of endothelial cells; this activity is suggested to be dependent on EPAC1, an exchange factor for the aforementioned small GTPase RAP-1, and VE-cadherin organization (unpublished findings). Altogether, these data on the molecular mechanisms and more general functional effects of LMWF5A demonstrate how this biologic may prove useful for the treatment of lung vasogenic edema by enhancing the alveolar-epithelial barrier via altering GTPase activity, promoting cortical f-actin rearrangement, and stabilizing the microtubule network.

### Testing of hypothesis

To explore the use of LMWF5A for this indication, a randomized controlled trial to evaluate the safety and efficacy of nebulized LMWF5A in adults with ARDS secondary to COVID-19 infection (supplement 1) was developed and is under review by the Center for Biologics Evaluation and Research division of the Food and Drug Administration. Briefly, this trial is designed to enroll up to ten (10) patients, randomized 1:1 to nebulized LMWF5A plus standard of care (SOC) for ARDS (active arm, *n* = 5) or SOC for ARDS (control arm, n = 5). The primary trial objective is to evaluate the safety and tolerability of nebulized LMWF5A in patients with ARDS secondary to COVID-19 infection. The secondary trial objectives are to evaluate the efficacy of nebulized Ampion versus control in improving the clinical course and outcomes of patients with ARDS secondary to COVID-19 infection including mortality, ventilator free days and PaO2/FiO2 ratio.

### Implications of the hypothesis

If successful, this therapy may attenuate the cytokine storm observed in these patients and potentially reduce mortality, increase ventilation free days, improve oxygenation parameters and consequently lessen the burden on patients and the intensive care unit. This initial study will be followed by larger randomized controlled trial in COVID-19 positive patients exhibiting respiratory distress and might avoid the need of mechanical ventilation.

## Conclusion

In conclusion, in vitro findings suggest that the immunomodulatory effects of LMWF5A make it a viable candidate for treating cytokine storm and restoring homeostasis to the immune response in COVID-19.

## Data Availability

The data analyzed during in this report are included in published articles or available from the corresponding author on reasonable request.

## Abbreviations

LMWF5A: Low molecular weight fraction of human serum albumin
DA-DKP: Aspartyl-alanyl diketopiperazine
SARS-CoV-2: Severe acute respiratory syndrome coronavirus 2
SARS-CoV: Severe acute respiratory syndrome coronavirus
COVID-19: Coronavirus disease of 2019
ARDS: Acute respiratory distress syndrome
ALI: Acute lung injury
LPS: Lipopolysaccharide
DAMP: Damage-associated molecular pattern
PAMP: Pathogen-associated molecular pattern
PBMC: Peripheral blood mononuclear cells
BMMSC: Bone marrow derived mesenchymal stem cells
PMA: Phorbol 12-myristate 13-acetate
PRR: Pattern recognition receptor
TLR: Toll-like receptor
HA: Influenza Hemagglutinin
TNFα: Tumor necrosis factor alpha
IFNγ: Interferon gamma
IL-1β: Interleukin 1 beta
IL-1Ra: Interleukin 1 receptor antagonist
IL-6: Interleukin 6
IL-10: Interleukin 10
IL-12: Interleukin 12
IL-21: Interleukin 21
IP10 or CXCL10: C-X-C Motif Chemokine Ligand 10
NF-κB: Nuclear factor kappa-light-chain-enhancer of activated B cells
p50: NF-κB subunit p50
RelA: NF-κB subunit p65
RelB: NF-κB subunit RelB
STAT: Signal transducer and activator of transcription
AhR: Aryl hydrocarbon receptor
PPAR: Peroxisome proliferator-activated receptor
COX-2: Prostaglandin-endoperoxide synthase 2
PGE2: Prostaglandin E_2_
PGD2: Prostaglandin D_2_
15d-PGJ2: 15-Deoxy-Δ-12,14-Prostaglandin J_2_
GTPase: Guanosine triphosphate hydrolase
RAP-1: Ras-related protein RAP-1
RhoA: Ras homolog family member A
Rac1: Rac family small GTPase 1
EPAC: Exchange protein activated by cAMP
